# Chunking or not chunking? How do we find words in artificial language learning?

**DOI:** 10.2478/v10053-008-0111-3

**Published:** 2012-05-21

**Authors:** Ana Franco, Arnaud Destrebecqz

**Affiliations:** Cognition, Consciousness, and Computation Group, Université Libre de Bruxelles, Belgium

**Keywords:** implicit statistical learning, transitional probabilities, chunking, serial reaction, time task

## Abstract

What is the nature of the representations acquired in implicit statistical learning? Recent results in the field of language learning have shown that adults and infants are able to find the words of an artificial language when exposed to a continuous auditory sequence consisting in a random ordering of these words. Such performance can only be based on processing the transitional probabilities between sequence elements. Two different kinds of mechanisms may account for these data: Participants may either parse the sequence into smaller chunks corresponding to the words of the artificial language, or they may become progressively sensitive to the actual values of the transitional probabilities between syllables. The two accounts are difficult to differentiate because they make similar predictions in comparable experimental settings. In this study, we present two experiments that aimed at contrasting these two theories. In these experiments, participants had to learn 2 sets of pseudo-linguistic regularities: Language 1 (L1) and Language 2 (L2) presented in the context of a serial reaction time task. L1 and L2 were either unrelated (none of the syllabic transitions of L1 were present in L2), or partly related (some of the intra-words transitions of L1 were used as inter-words transitions of L2). The two accounts make opposite predictions in these two settings. Our results indicate that the nature of the representations depends on the learning condition. When cues were presented to facilitate parsing of the sequence, participants learned the words of the artificial language. However, when no cues were provided, performance was strongly influenced by the employed transitional probabilities.

## Introduction

When faced with a complex structured domain, human learners tend to behave as if they extract the underlying rules of the material. In an artificial grammar learning experiment, for instance, participants are first requested to memorize a series of letter strings following the rules of a finite-state grammar. They are not informed of the existence of those rules, however. In a second phase of the experiment, when asked to classify novel strings as grammatical or not, they usually perform above chance level but remain generally unable to verbalize much of the rules. Such a dissociation has been initially attributed to the unconscious or implicit learning of the underlying rules (Reber, 1967, 1989).

This interpretation has ever since been heavily debated, however. What is the exact nature of learning? Is performance based on learning the abstract rules of the material or on the surface features of the training items, such as the frequencies of individual elements or chunks? Most recent implicit learning studies suggest that this latter view provides a better account of performance(e.g., Perruchet & Pacteau, 1990). Several experiments have indeed demonstrated that performance was based on “fragmentary” learning. In other words, learning would depend on the memorization of fragments of the stimuli presented to the subjects instead of on an abstract rule-extraction process (Meulemans & Van der Linden, 1997; Perruchet & Amorim, 1992; Perruchet & Pacteau, 1990; E. Servan-Schreiber & Anderson, 1990).

Over the last few years, a series of experimental results have provided new insights into the question of the nature of the representations involved in implicit learning. Research on language acquisition has indeed shown that 8-months old infants are sensitive to statistical information (Jusczyk, Luce, & Charles-Luce, 1994; Saffran, Aslin, & Newport, 1996; Saffran, Johnson, Aslin, & Newport, 1999) and capable of learning distributional relationships between linguistic units presented in the continuous speech stream formed by an artificial language (Gomez & Gerken, 1999; Jusczyk, Houston, & Newsome, 1999; Perruchet & Desaulty, 2008; Saffran et al., 1996). The seminal studies by Saffran and collaborators have shown that infants, children, and adults were able to find the “words” from an artificial language when presented with a concatenation of those plurisyllabic sequences (e.g., *batubi*, *dutaba*) presented in a random order and forming a continuous stream without any phonological or prosodic markers. Only the transitional probabilities (TPs) between syllables can be used to discover the word boundaries. Indeed, as the next word in the stream can never be anticipated, those TPs are stronger intra-word than between words.

Other studies have indicated that these mechanisms are not restricted to linguistic material but also apply to auditory non-linguistic stimuli (e.g., Saffran et al., 1999) or to visual stimuli (e.g., Fiser & Aslin, 2002; Hunt & Aslin, 2001). In the same way, implicit sequence learning studies have indicated that human learners are good at detecting the statistical regularities present in a serial reaction time (SRT) task. Altogether, these data suggest that statistical learning depends on associative learning mechanisms picking up the input’s statistical constraints rather than on the existence of a “rule abstractor device” (Perruchet, Tyler, Galland, & Peereman, 2004).

Different computational models have been proposed to account for the data. On the one hand, according to the simple recurrent network model (SRN; Elman, 1990; see also Cleeremans, 1993; Cleeremans, & McClelland, 1991), learning is based on the development of associations between the temporal context in which the successive elements occur and their possible successors. Over training, the network learns to provide the best prediction of the next target in a given context, based on the transitional probabilities between the different sequence elements. On the other hand, models such as PARSER (Perruchet & Vinter, 1998), consider learning as an attention-based parsing process that results in the formation of distinctive, unitary, rigid representations or chunks. In contrast with the SRN, PARSER finds and stores the most frequent sequences in memory files or mental lexicon. Thus, both models are based on processing statistical regularities, but only PARSER leads to the formation of “word-like” units.

In a recent paper, Frank, Goldwater, Griffiths, and Tenenbaum (2010) classified the SRN and PARSER as examples of transition-finding or chunking models, respectively. The first model implements a bracketing strategy, according to which participants are assumed to insert boundaries into the sequence of speech. The second model implements a clustering: strategy that consists in grouping certain speech sequences together into units (Giroux & Rey, 2009; Swingley, 2005).

Although the processes and representations assumed by these two classes of models are quite different, contrasting their assumptions is difficult as they make similar predictions in most experimental settings. For instance, in an artificial language learning experiment (including the pseudowords *batubi* and *dutaba*), as the representations that emerge in either model reflect the strength of the associations between sequence elements, both predicted improved processing of intra-words (e.g., *ba-tu*) than inter-words transitions (e.g., *bi-du*) as well as successful recognition of the words of the artificial language (Saffran, Newport, Aslin, Tunick, & Barrueco, 1997). Besides, as noted by Perruchet and Pacton (2006), researchers in statistical learning tend to acknowledge the existence of chunk-like representations. Jenny Saffran (2001) showed, for instance, that presented with an artificial language, 8-month infants develop word-like representations rather than merely probabilistically-related sequences of sounds.

Some recent studies were conducted to distinguish the two models. In a recent experiment, Giroux and Rey (2009) compared lexical and sublexical recognition performance of adults after hearing 2 or 10 min of an artificial spoken language. A *sublexical unit*, or *part-word*, is a sequence of syllables composed of the end of a word and of the beginning of another word. They found that, as predicted by PARSER but not by the SRN, part-words recognition performance did not increase with longer exposure and that performance on words was better than performance on part-words only after 10 min.

In another study, Endress and Mehler (2009) presented participants with an artificial language containing three syllable-words. Each of these words was generated by modifying one syllable of what they called a phantom word that was never actually presented during the experiment. Endress and Mehler observed that after exposure participants preferred words to part-words containing low-frequency transitions but that they tended to consider phantom-words as words of the artificial language. They indeed failed to prefer words to phantom-words. This remained true even after arbitrarily long exposure phases. Importantly, participants also preferred *phantom words* to part-words even when these latter sequences were more frequently presented during the learning phase. The authors concluded that computing TPs is not sufficient for the extraction of word-like units and that other cues have to be processed for speech segmentation to occur (see Perruchet & Tillmann, 2010, for a recent discussion on that topic).

Finally, a recent study in the visual domain (Orbán, Fiser, Aslin, & Lengyel, 2008) provided further arguments in favor of the chunking hypothesis. In that study, participants learned scenes or assemblies of visual shapes statistically organized in pairs. They were then presented with two partial scenes and had to select the test scene more familiar based on the scenes viewed during familiarization. One test item was a combination of shapes from the training phase (or a part thereof, called an *embedded combination*) and the other test item consisted of shapes from two different pairs (a *mixture combination*). Orbán et al. devised an experiment that contained two sets of four shapes in which both the first- (frequency of shapes) and second-order statistics between shapes (frequency of pairs) were made identical. However, the shapes in one of the sets were always shown as triplet combos, whereas the shapes in the other group were shown individually (and occasionally all four of them were presented together). Results in the test phase indicated that participants formed chunks in the first but not in the second group of shapes. Indeed, they were able to recognize triplets from the first group of four against mixture triplets, they were also able to distinguish between triplets constructed from the elements of the two groups in a direct comparison but they did not make the distinction between triplets constructed from the shapes of the second group of four with mixture triplets.

Orbán et al. (2008) compared human performance to the performance of two computational models: (a) an associative-learner (AL) that learns pair-wise correlations between shapes without an explicit notion of chunks and (b) a chunking model implementing bayesian learning processes (BCL). Consistent with human performance, the BCL successfully learned to distinguish between triplets constructed from the elements of the two groups of four shapes, whereas the AL was not able to make this distinction. As human participants, both the BCL and the AL correctly recognized triplets from the first group of four shapes against mixture triplets. Unlike human participants, the AL (but not the BCL) falsely recognized triplets constructed from the shapes of the second group of four against mixture triplets.

To sum up, the available evidence in the auditory and visual domains suggests that chunking models provide a better account of human statistical learning abilities. These results are in contrast with the sequence learning literature. In that domain, several studies have shown that human performance could be accurately accounted for by the mere associative learning implement in the SRN (Cleeremans, 1993; Cleeremans & McClelland, 1991; D. Servan-Schreiber, Cleeremans, & McClelland, 1991). Specifically, the SRN has been proved to be able to reproduce RT learning curves in sequence learning studies even though it does not form chunk-like representations. The SRN is a connectionist network, level of activation at the output level of the network are considered as preparation for the next sequence event. To account for performance, the SRT task is viewed as a prediction task, and high activation levels correspond to faster reaction times (RTs). As Cleeremans and McClelland have shown, with training, the pattern of activation at the output level will more and more precisely represent the transitional probabilities between any two sequence elements. To account for recognition performance, the average output activation is computed when a small fragment sequence is presented to the network. In a two-alternative forced-choice (2AFC) task, the sequence fragment producing the more activation at the output level would be considered as recognized or familiar.

A model such as PARSER by contrast does not have a direct way to simulate RTs, but it can easily account for performance in a recognition or familiarity judgment task. When trained with an artificial language, a random parameter between 1 and 3 determines at each time step the number of elements (e.g., syllables) processed simultaneously by PARSER and stored as a new representational unit of the perceptual memory. Each of these new units receives an initial weight. The weights of the units increase each time they are processed again or decrease on each processing cycle. The value of the decrement depends on the forgetting and interference parameters. There is a threshold above which a given unit shapes perception.

In a recognition trial in a 2AFC task, the response of the model will depend on the units stored in the perceptual shaper. If the units corresponding to the two test items are both represented in the perceptual shaper, the response of the model will correspond to the unit with the strongest weight. If only one item is represented, it will correspond to the model’s response. If none of the items is represented, the model’s choice is determined randomly (see, e.g., Giroux & Rey, 2009).

In the next section, we will describe how we contrasted the predictions of these two models in the context of a choice RT task implementing the statistical regularities of an artificial language similar to those used by Saffran and collaborators (e.g., Saffran et al., 1999). We did not run simulations but conducted two experiments in which different predictions can be made according to a chunking model such as PARSER or a transition-finding model such as the SRN.

## Overview of the experiments

**Figure 1.**
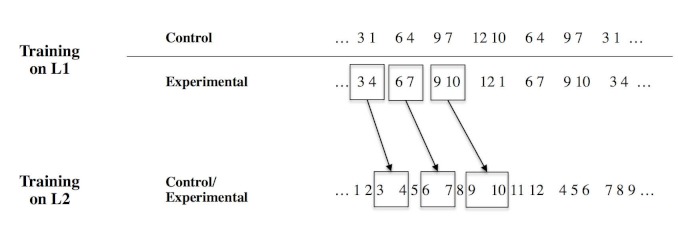
Percentage of participants categorized as “verbalizers” in the five experimental groups with randomized and systematic training. In the mI n the control condition, Language 1 (L1) and Language 2 (L2) are unrelated. In the experimental condition, some of L1 “intra-word” transitions become L2 “intra-word”.

To contrast the predictions of chunking and transition-finding strategies, we used a 12-choice SRT task in which the succession of the visual targets implemented statistical regularities similar to those found in artificial languages. We choose to use a visuomotor task instead of presenting the artificial language in the auditory modality in order to be able to track the development of statistical learning through reaction times (see Misyak, Christiansen, & Tomblin, 2010, for a recent similar attempt; see also Conway & Christiansen, 2009, for a systematic comparison between the auditory and visual modalities). In our version of the task, participants had to learn two different artificial languages presented successively. In our experiments, the first “language” (L1) was composed of four “words”, or small two-element sequences, and the second “language” (L2) was composed of four small three-element sequences. In one (control) condition, the two ensembles were not related to each other, but in the other (experimental) condition, the intra-sequences transitions of L1 became inter-sequences transitions in L2 (see Figure 1 and Table 1.).

**Table 1.** The Four Three-Element Sequences Used During Language 1 (L1) and Language 2 (L2) Training in the Control and Experimental Conditions.

	L1	L2
Control	Experimental	Control and experimental
3-1	3-4	1-2-3
6-4	6-7	4-5-6
9-7	9-10	7-8-9
12-10	12-1	10-11-12

*Note*. L1 differs between the control and experimental condition but L2 is the same in the two conditions.

The SRN and PARSER would predict two different outcomes in this situation. Namely, these two models makes distinct predictions regarding the way L1 learning influences L2 learning as indexed in the L2 sequences recognition performance in the 2AFC task. However, both chunking and associative processes would predict faster RT within than between sequences as performance can be improved either because the transitional probability between two sequence elements is high or because these two sequence elements are part of the same chunk.

The probability that one sequence element follows another in the input stream is 100% within-words and 33% between-words (since there are four different sequences and no repetitions). After a sufficient amount of training, a system such as the SRN will learn these transitional probabilities so that it would perfectly predict the Element 1 when presented with the input Element 3. When switching from L1 to L2, a transition-learner will thus have to develop new associations between elements in the control condition. For instance, while “3” was only associated with “1” in L1, the system will learn to predict “4,” “7,” or “10” after the presentation of “3” when presented with L2 because the sequence “123” could be followed by “456”, ”789” or “101112”. In the experimental condition, after training on L1, that comprises the sequence “34”, the system will have simply to “tune” the strength of the association between “3” and “4” as it is only 33% in L2 and not 100% as it was in L1.

Turning now to the chunking process, recall that in PARSER, each element is associated with the other elements of the same chunk but there is no association whatsoever with the other elements. In other words, there is a within-chunk strength of 100% and a between-chunk strength of 0%. Due to the interference and forgetting parameters, the formed representational units will progressively vanish unless they are presented again in the input stream. At some point, the content of the perceptual memory will correspond to the largest possible chunks that could be extracted from the input sequence. At that point, a given element is included in one chunk only. For instance, if the Element 3 is part of the chunk “31”, it cannot be also associated with other elements in order to form a chunk “123”. As a consequence, when presented with L2, the learning system will first have to break the chunks formed during training on L1 in order to form the new L2 chunks. This task should be easier in the control than in the experimental group since, in the former case, L1 transitions are no longer presented during L2. L1 chunks will then progressively decay and be replaced by L2 chunks. By contrast, in the experimental condition, L1 transitions are still presented, although less frequently, between L2 sequences. As a result, L1 chunks continue to be reinforced during L2 presentation. It will then be more difficult for a chunking system to learn L2 after L1 in the experimental condition. If human learning is based on similar chunking processes, one might therefore expect better recognition of L2 sequences in the control than in the experimental condition.

Another prediction concerns recognition performance with “part-sequences” (i.e., three-element chunks that span over a transition between two sequences). In the test phase, participants were presented with three types of test items (see Table 2.), three-element sequences of L2, “non-sequences” of L2 (three-element sequences involving transitions that were never presented in the exposition phase), and “part-sequences” (involving one transition that was part of a L2 sequence and one “between-sequence” transition of L2). If learning is based on transitional probabilities, participants may find it more difficult to exclude part-sequences than non-sequences as, on average, the association strength between sequence elements is higher in the former than in the latter cases. By contrast, if performance is based on chunking processes similar to those of PARSER, participants should exclude part-sequences as easily as non-sequences as, in both cases, these items are not part of the perceptual memory.

**Table 2.** The Test Items Used in Experiments 1 and 2.

Test items		
L2 sequences	Part- L2 sequences	Non- L2 sequences
1-2-3	3-7-8	1-4-7
4-5-6	6-10-11	3-11-8
7-8-9	9-1-2	5-2-10
10-11-12	10-4-5	6-12-9

*Note*. L1 = Language 1. L2 = Language 2.

## Experiment 1

The goal of Experiment 1 was twofold. First, we wanted to make sure that participants could learn statistical regularities similarly as those used in artificial languages in the context of a SRT task. Second, we wanted to establish whether they will be able to recognize the L2 “words”, that is, the three-element sequences presented in a random order during the SRT task. If learning is based on chunking, recognition performance should be the same for non-sequences and part-sequences. If performance is based on learning transitional probabilities, participants may more frequently consider part-sequences than non-sequences as L2 sequences. The chunking hypothesis also predicts better L2 sequence-recognition in the control than in the experimental condition.

### Method

#### Participants, apparatus, and stimuli

Twelve undergraduate students (eight female and four male; *M*_age_ = 20.9) of the Université Libre de Bruxelles took part in the experiment in exchange for course credits. All reported normal or corrected-to-normal vision. This experiment was approved by the Ethics Committee of the Faculté des Sciences Psychologiques et de l’Education (Faculty of Psychology and Education) of the Université Libre de Bruxelles.

The experiment was run on a Mac mini computer equipped with a touch sensitive screen monitor. The display consisted of 12 invisible dots arranged in a square on the computer’s screen. Each dot represented a possible position of the visual moving target. The stimulus was a small red circle 0.65 cm in diameter that appeared on a gray background, centered 0.10 cm below one of the 12 invisible dots separated by 2.20 cm.

The stimulus set consisted of sequences of visual locations in which the visual target could occur on one out of 12 different positions (numbered from 1 to 12, see Table 1.). In the control condition, L1 contained four two-location sequences: “31”, “64”, “97”, and “1210”. In the experimental condition, the sequences were “34”, “67”, “910”, and “121”. In both conditions, L2 contained four three-location sequences: “123”, “456”, “789”, and “101112”. The stimuli were presented in a pseudo-random order: a sequence was never directly repeated. A different mapping between the 12 sequence elements and the 12 screen locations was used for each participant.

#### Procedure

The experiment consisted of nine training blocks during which participants were exposed to two different language-like sequences in a SRT task. In the first three training blocks, they were exposed to a first language (L1) composed by four two-location “words” or sequences (see Table 1.). Each sequence was presented 200 times, for a total of 1,600 trials. In the six subsequent blocks, participants were exposed to a second language (L2) composed by four three-location sequences presented 250 times each, for a total of 3,000 trials. L2 exceeded L1 training in order to make sure that the second language that would further be tested in a 2AFC task, was learned. On each trial, a stimulus appeared at one of the 12 possible positions. Participants were instructed to press the location of the target as fast as possible with the ad hoc pen. The target was removed as soon as when it had been pressed, and the next stimulus appeared after either a 250 ms response-stimulus interval (RSI) for intra-sequences transitions or a 750 ms RSI for inter-sequence transitions. Participants were not informed that the sequence of locations corresponded to the succession, in a random order, of the four sequences of the artificial languages. They were allowed to take short rest breaks between blocks.

Participants were randomly assigned to two conditions. In the experimental condition, one third of the inter-sequences transitions of L2 were identical to L1 sequences (see Figure 1). This was not the case in the control condition in which L1 and L2 were unrelated. L1 differed between control and experimental conditions whereas L2 was the same in both conditions. In the experimental condition, the intra-sequence transitions of L1 became inter-sequences transitions in L2. For example, the L2 sequence “123” is followed by “456” in one third of the cases. In that case the inter-sequence transition “34” corresponds to a L1 sequence.

All participants subsequently performed a recognition task in which they had to decide whether they had been exposed to each sequence during the training phase or not. Three types of sequences were presented (see Table 2.): the sequences from L2 (each sequence presented twice); four part-sequences, that is, sequences composed by the end of a sequence and the beginning of another sequence; and four non-sequences, corresponding to visual sequences which had never been presented during L2 training. In total, the experiment lasted approximately 50 min.

### Results

#### Reaction time results

**Figure 2.**
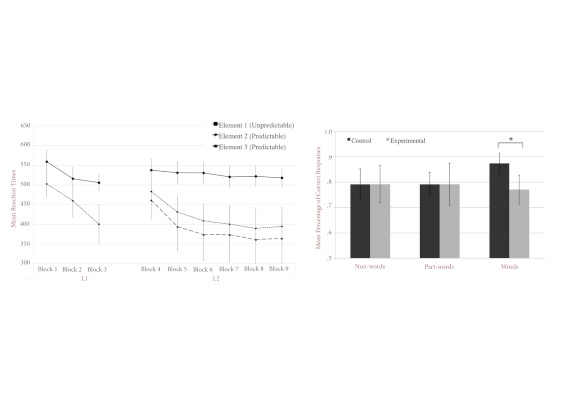
The figure shows mean reaction times (RTs) obtained for unpredictable (Element 1) and predictable elements (Elements 2 and 3) during Language 1 (L1) and Language 2 (L2) blocks. RTs are averaged over experimental and control conditions (left panel). Mean percentage of correct responses during the recognition task for words, non-words, and part-words in the control and experimental conditions are displayed on the right panel. Chance level = 50%.

To assess whether participants were able to learn L1 and L2, we examined separately mean RTs for the first three blocks (L1) and for the next six blocks (L2) in the control and experimental conditions. Recall that the stimulus material was such that the first element of each sequence was unpredictable, whereas the second element (and third element in L2) were completely predictable. Figure 2 (left panel) shows the average RTs obtained over the entire experiment, plotted separately for each element of the sequences. Given that participants performed similarly in the control and in the experimental conditions, *F*(1, 10) = 2.113,*p* > .1, for L1; and *F*(1, 10) = 0.481, *p* > .5, for L2, we pooled them together. It appeared that participants’ responses were strongly influenced by the serial position within each sequence: RTs decreased more and were faster for predictable elements than for unpredictable elements (cf. Figure 2). Two two-way analyses of variance (ANOVA) conducted on mean RTs confirmed these impressions. First, we examined the first three blocks (L1) by using an ANOVA with Block (3 levels) and Element (2 levels - predictable and unpredictable) as repeated measures factors. This analysis revealed a significant main effect of block, *F*(2, 10) = 56.007, *p* < .0001, partial η^2^ = .804; and element, *F*(1, 10) = 15.431,*p* < .005, partial η^2^ = .520. The interaction also reached significance, *F*(2, 10) = 6.630, *p* < .01, partial η^2^ = .399. Second, we examined the next six blocks (L2) by using an ANOVA with Block (6 levels) and Element (3 levels) as repeated measures factors. A significant main effect of block was found, *F*(5, 50)= 15.113, *p* < .0001, partial η^2^ = .592.The analysis also revealed a significant main effect of element, *F*(2, 20) = 25.141, p < .0001, partial η^2^ = .707. The interaction also reached significance, *F*(10, 100) = 6.220, *p* < .0001, partial η^2^ = .377.

#### Recognition task results

Figure 2 (right panel) shows recognition performance for the three types of test sequences plotted separately for control and experimental conditions. Inspection of the figure indicates that the participants recognized L2 sequences, non-sequences, and part-sequences in the two conditions.

In order to ensure that the sensitivity of the recognition measure is independent of response bias, that is, is not affected by the participants’ own report criteria, we used the signal detection theory in the same way as in Tunney and Shanks (2003). For each participant, we computed a *d*’ value reflecting the ability to discriminate between old and new sequences. Hits correspond to “yes” responses to old triplets - (correct responses), and false alarms correspond to “yes” responses to new sequences - (incorrect responses). A one sample t-test on the *d*’ distribution indicates that, on average, participants were able to discriminate between old and new triplets, mean *d*’ = 3.022, *t*(11) = 4.567 *p* = .001. Performance was above chance level in both conditions and for each type of test triplet as confirmed by a series of one-tailed t-tests (see Table 3.).

**Table 3.** *t* Values Comparing Recognition Scores to Chance Level in Control and Experimental Conditions for the Three Types of Test Sequences.

	Words	Non-words	Part-words
Control	5,82*	2,91*	3,79*
Experimental	2,89*	2,44*	2,15*

*p* < .05 (one-tailed).

More importantly, performance was reliably better for L2 sequences in the control condition as compared to the experimental condition, *t*(47) = 1.70, *p* < .05 (one-tailed). All the other comparisons were not significant.

### Discussion

Our SRT results indicate that participants learned L1 and L2 in both experimental and control conditions. The recognition results showed that participants were able to discriminate the sequences of L2. Importantly, performance was improved in the control condition as compared to the experimental condition, that is, when the two language-like sequences did not share any transitions between elements. Taken together, these results are in line with the notion that participants learned the sequences based on parsing mechanisms.

Recall that, in the experimental condition, L1 transitions (e.g., “34”) were still presented between sequences during L2 presentation (e.g., between “123” and “456”). As a result, L1 chunks continue to be reinforced during L2 presentation. As a consequence, a chunking model, such as PARSER for instance, would predict better L2 recognition in the control than in the experimental condition. Indeed, it should be more difficult for such a model to develop new representations for the new L2 sequences if the previous, conflicting representations developed during L1 were still reinforced.

The observation that non-sequences and part-sequences rejection did not differ between the two conditions also fits with the prediction of a chunking model. The representational units that result from learning in such a model do not reflect the actual transitional probabilities present in the training sequence. The probability to erroneously consider a test sequence as a sequence of L2 should then not be higher for part-sequences than for non-sequences even though the transitional probabilities are, on average, higher in the former cases.

In Experiment 1, however, sequences were clearly identified by the use of a larger RSI for inter-sequences than for intra-sequence transitions. It remains therefore possible that our results depend on this particular presentation mode. In other words, learning would fit with chunking models simply because the input stream was already parsed into consistent chunks. To address this possibility, we conducted a second experiment in which the RSI was set to a constant value.

## Experiment 2

### Participants, apparatus, stimuli, and procedure

Ten undergraduate students (six female, four male; *M*_age_ = 21.3) of the Université Libre de Bruxelles took part in the experiment in exchange for course credits. All reported normal or corrected-to-normal vision. This experiment was approved by the Ethics Committee of the Faculté des Sciences Psychologiques et de l’Education (Faculty of Psychology and Education) of the Université Libre de Bruxelles.

The apparatus and display were identical to those used in Experiment 1. The procedure was identical to the one used in Experiment 1except for the fact that the RSI was fixed at 250 ms for intra-sequence and inter-sequence transitions. The stimuli were identical to those used in Experiment 1. A different mapping was also used for each participant in this experiment even though a given sequence element was associated with only 10 out of the 12 possible screen locations.

### Results

#### Reaction time results

**Figure 3.**
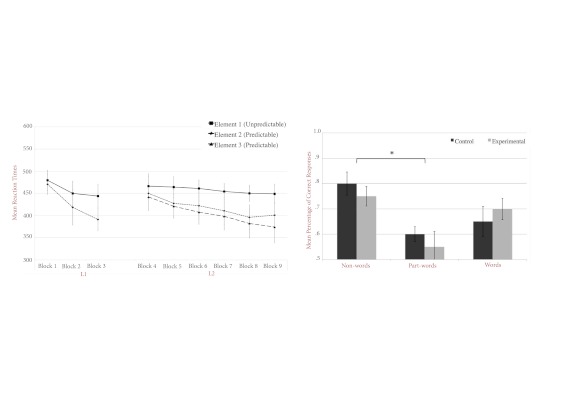
The figure shows mean reaction times (RTs) obtained for unpredictable (Element 1) and predictable elements (Elements 2 and 3) during Language 1 (L1) and Language 2 (L2) blocks. RTs are average over experimental and control conditions (left panel). Mean percentage of correct responses during the recognition task for words, non-words, and part-words in the control and experimental conditions are displayed on the right panel. Chance level = 50%.

Figure 3 (left panel) shows the average RTs obtained over the entire experiment, plotted separately for each element of the sequences. As in Experiment 1, control and experimental conditions were pooled together since there was no difference in performance between both conditions, *F*(1, 8) = 1.114, *p* >.1, for L1; and *F*(1, 8) = 0.042, *p* > .5, for L2. The results clearly indicate that RTs are strongly influenced by the position: RTs decreased more and were faster for predictable elements than for unpredictable elements.

Two two-way ANOVA conducted on mean RTs confirmed these impressions. First, we examined the first three blocks (L1) by using an ANOVA with Block (3 levels) and Element (2 levels - predictable and unpredictable) as repeated measures factors. This analysis revealed a significant main effect of block, *F*(2, 16) = 37.227, *p* < .0001, partial η^2^ = .807; and of element, *F*(1, 8) = 9.720, *p* < .05, partial η^2^= .525. The interaction also reached significance, *F*(2, 16) = 7.337, *p* < .005, partial η^2^ = .474. Second, we examined the next six blocks (L2) by using an ANOVA with Block (6 levels) and Element (3 levels) as repeated measures factors. We found a significant main effect of block, *F*(5, 40) = 9.657, *p* < .005, partial η^2^ = .490. The analysis also revealed a main effect of element, *F*(2, 16) = 8.404, *p* < .005, partial η^2^ = .472. The interaction also reached significance, *F*(10, 80) = 6.914, *p* < .0001, partial η^2^ = .437.

#### Recognition task results

To analyze recognition performance, we first computed a *d*’ value as in Experiment 1. A one-sample *t* test on the *d*’ distribution showed that participants were able to discriminate between old and new sequences, mean *d*’ = 1.150, *t*(9) = 3.035, *p* = .014. As indicated in Table 4., participants were able to correctly reject non-sequences in both conditions. They did not, however, correctly reject part-sequences. Concerning L2 sequences, experimental participants recognized them above chance but this was not the case in control participants.

**Table 4.** *t* Values Comparing Recognition Scores to Chance Level in Control and Experimental Conditions for the Three Types of Test Sequences.

	Words	Non-words	Part-words
Control	1,24	3,21*	1,63*
Experimental	2,36*	3,17*	0,41*

*p* < .05 (one-tailed).

Second, we analyzed the proportions of correct recognitions, plotted in Figure 3 (right panel), measured in the two conditions and for the three types of test sequences. Overall, performance did not significantly differ between control and experimental conditions (for all differences *p* > .05). Therefore, we pooled control and experimental conditions together and compared performance for non-sequences, part-sequences, and L2 sequences. This analysis revealed a significant difference between non-sequences and part-sequences, paired *t*(78) = 1.574, *p* < .05. Non-sequences were reliably more correctly rejected than part-sequences (see Figure 3, right panel). The other comparisons failed to reach significance.

### Discussion

In Experiment 2, L1 and L2 were presented using a constant RSI. As in Experiment 1, participants learned the first and second languages. Indeed, throughout training, mean RTs decreased more for predictable than for unpredictable elements. Moreover, participants recognized L2 sequences, at least in the experimental condition, and correctly rejected non-sequences. Interestingly, in both experimental and control conditions, participants performed better in rejecting non-sequences than part-sequences, which were not correctly rejected.

According to PARSER, performance should be the same for non-sequences and part-sequences. If participants formed L2 chunks during training, it should be as easy to reject non-sequences as part-sequences as these sequences do not match the units formed during training. On the contrary, the SRN predicts that participants should recognize L2 sequences, which correspond to high transitional probabilities, and reject non-sequences, which correspond to low transitional probabilities. However, as part-sequences involved high transitional probabilities, the SRN may have more difficulties in rejecting them. The results of Experiment 2 nicely fit with the SRN predictions, suggesting that participants are indeed sensitive to the actual values of the transitional probabilities between sequence elements. When considering Experiments 1 and 2 together, our results suggest that the values of transitional probabilities influence performance when temporal cues do not guide the chunking process.

## General Discussion

In this paper, we aimed at clarifying the nature of the representations involved in implicit and statistical learning. The question was to assess whether participants form chunks of the training material or merely develop a sensitivity to the transitional probabilities present in the training sequence. In line with previous studies showing that statistical learning of pseudolinguistic regularities can occur in other modalities than the auditory modality, we showed, in the context of a visuo-motor RT task, that participants learn the statistical regularities present in a random succession of sequences of visual targets. The RT results indicate that participants were able to learn two different languages (L1 and L2) presented successively. Moreover, they were also able to recognize L2 sequences in a subsequent recognition task.

When sequences were clearly separated from each other in Experiment 1, recognition performance was improved in a control condition in which L1 and L2 did not share any pairwise transitions between sequence elements. These results are in line with the notion that word-like, rigid, disjunctive units are developed during learning. However, chunk formation seems not to be automatic in our task. When sequences were not clearly identified in Experiment 2, that is, when they were presented in a continuous stream without any temporal cue to guide the chunking process, recognition performance was more strongly affected by the actual values of the transitional probabilities between sequence elements. This was reflected in Experiment 2 by better rejection of non-sequences than part-sequences in the recognition task. This pattern of results is in line with previous studies showing that the temporal distribution of the input affects statistical learning in the visual modality (Conway & Christiansen, 2009).

How are these findings related to natural languages segmentation? A large body of evidence indicates that, in the absence of a clear word-boundary cue in the signal, word segmentation in natural language is based on lexical, sublexical, phonetic, phonotactic, and prosodic cues (Mattys, Jusczyk, Luce, & Morgan, 1999). Research on natural speech indicates that lower level, signal-contingent cues will be more prone to influence segmentation when the availability of higher level lexical information decreases (Mattys, White, & Melhorn, 2005). In the same way, our results suggest that in the absence of a clear temporal cue, recognition performance is more affected by the strength of the transitional probabilities as it is the only available cue to find between-sequences boundaries.

In line with a modality-constrained view of implicit statistical learning (Conway & Pisoni, 2008), previous studies have shown that statistical learning was differentially affected by training conditions in the auditory and visual modalities (Conway & Christiansen, 2005, 2008; Saffran, 2002). The rate of presentation of the input stream, for instance, influences more the statistical learning of sequential regularities in the visual than in the auditory modality. Our results also suggest that the nature of the statistical learning processes involved in our visuo-motor task could be modulated by the rate of presentation of the sequence of visual targets. Participants chunk the sequence according to the “words” of the artificial language when they are clearly marked by the temporal structure of the input, not otherwise.

Another possible explanation for this result could be that participants did form chunks in Experiment 2 but not those that corresponded to the actual L2 sequences. It is possible that participants indeed parsed the continuous sequence of visual stimuli into smaller chunks but that these chunks did not respect the actual boundaries between L2 sequences. It is also not necessarily the case that all sequential transitions end up as being part of a larger chunk. Participants may have focused, for instance, on particularly salient transitions (e.g., between elements that were spatially close to each other or between alternating locations) and end up with larger, smaller, or different chunks than those corresponding to the sequences of the artificial language. In other words, if chunking is not directly induced by the presentation mode, attentional factors may also influence chunk formation. As a consequence, the actual chunks may differ from one participant to another and may not strictly reflect the transitional probabilities between the different sequence elements. This may, of course, influence recognition performance as a different parsing from one participant to another would tend to cancel each other out.

Both the SRN and PARSER implement elementary associative learning mechanisms such that, in both cases, the system tends to associate elements that occur often in succession. As a consequence, even if the chunks resulting from training do not correspond to the actual sequences of the artificial language, there is a good chance that they involve highly frequent transitions. Participants may therefore tend to erroneously consider these part-sequences as sequences of the artificial language because they involve such high-frequency transitions.

It remains therefore possible that participants were not sufficiently trained on L2 in Experiment 2 in order to form the correct chunks of the second language. Recognition performance would then reflect intermediate chunk formation and these intermediate representations necessarily correspond more to part-sequences than to the never presented non-sequences.

Even though we cannot strictly exclude the possibility that chunks were formed in Experiment 2, recent sequence learning results suggest however that chunking does not take place when a cue inducing specific segmentation is removed. Jiménez, Méndez, Pasquali, Abrahamse, and Verwey (2011) also addressed the notion that chunking could be the main learning mechanism underlying sequence learning. They proposed a new index to capture segmentation in learning, based on the variance of responding to different parts of a sequence. They reasoned that discontinuous performance (indicating chunking processes) could be revealed through the observation of an increase in RT variance. Indeed, as participants should respond faster to sequence elements within- than between-chunks, RT variance should increase over training if learning is based on a growing number of chunks. As predicted, Jiménez et al. observed that participants who were induced to parse the sequence in a uniform way by using color cues responded much faster to the trials internal to a chunk than to those corresponding to the transition between successive chunks. By contrast, when the color cues were removed in a transfer phase, they did not respond faster to within-chunk transitions anymore. As a matter of fact, they did not respond differently from control participants who were not trained to chunk by colors beforehand. In line with our study, Jiménez et al. concluded that chunk learning arises when induced by a salient cue (the RSI in our study, the color of the stimulus in Jiménez et al.) while statistical learning of transitional probabilities occurs in more implicit settings, and in the absence of such salient cues.

Another central debate in the literature concerns the degree to which statistical learning depends upon specialized processes, devoted to the purpose of finding word-boundaries (domain-specific processes) or whether it is based on domain-general mechanisms dedicated to statistical computations. This issue is mostly discussed in the field of developmental psychology where the question is to know whether infants’ cognition is best viewed as the deployment of innate skills (Carey, 1999) or whether more weight should be put on the potential role of environmental structure in guiding development (Kirkham, Slemmer, & Johnson, 2002). In this view, the initial state would be better characterized by domain-general mechanisms that would adapt themselves to different types of input in different modalities (Dawson & Gerken, 2009; Karmiloff-Smith, 1992). That question is clearly beyond the scope of this study but our results suggest that learning can indeed be “tuned” to the input’s properties.

Finally, as mentioned before, statistical learning has been initially demonstrated in infants presented with a continuous stream of syllables (Saffran et al., 1996). Previous studies have also shown that infants and children were able to learn the statistical regularities present in a sequence of movements of a visual object (Kirkham, Slemmer, Richardson, & Johnson, 2007) or within a sequence of different visual shapes (Kirkham, Slemmer, & Johnson, 2002). Future work is needed, however, in order to measure whether the representational format of the acquired knowledge may also differ in infancy depending on training conditions.

In summary, this study suggests that when units are marked by a temporal cue, the chunking models provide reliable assumptions concerning the nature of the representations developed during learning. However, in the absence of cues guiding the chunking processes, performance appears to reflect the sensitivity to the strength of the transitional probabilities. This study suggests that prediction-based and clustering processes are not necessarily mutually exclusive but could be differentially associated with performance depending on training conditions.
